# 

**Published:** 1889-04-27

**Authors:** 


PRICE ONE PENNY.

No. 135, Vol. VI.—April 27th, 1889.

[Registered at the General Post Office as a Newspaper.]

Per Annum, 6s. 6d., post free.
Monthly Parts, 6d.; post free, 7½d.
Ditto per Annum, 7s. 6d., post free.

